# Inflammation and Fibrosis in Sleep-Disordered Breathing after Acute Myocardial Infarction

**DOI:** 10.3390/biomedicines12010154

**Published:** 2024-01-11

**Authors:** Jan Pec, Stefan Buchner, Henrik Fox, Olaf Oldenburg, Stefan Stadler, Lars S. Maier, Michael Arzt, Stefan Wagner

**Affiliations:** 1 Department of Internal Medicine II, University Hospital Regensburg, 93053 Regensburg, Germanylars.maier@ukr.de (L.S.M.); michael.arzt@ukr.de (M.A.); stefan.wagner@ukr.de (S.W.); 2 Department of Internal Medicine, Cham Hospital, 93413 Cham, Germany; 3 Clinic for General and Interventional Cardiology/Angiology, Heart and Diabetes Center NRW, Ruhr University Bochum, 32545 Bad Oeynhausen, Germany; 4 Center for Cardiology, Ludgerus-Kliniken, 48153 Münster, Germany; o.oldenburg@alexianer.de

**Keywords:** procollagen III type aminoterminal propeptide, sleep-disordered breathing, acute myocardial infarction, myocardial fibrosis, inflammation

## Abstract

Background: After acute myocardial infarction (AMI), inflammatory processes promote tissue remodeling at the infarct site. Procollagen III amino-terminal propeptide (PIIINP) is a circulating biomarker of type III collagen synthesis that has been shown to be associated with changes in left ventricular ejection fraction (LVEF) and predicts the occurrence of heart failure after AMI. We hypothesize that sleep-disordered breathing (SDB) promotes inflammation and myocardial fibrosis, leading to reduced myocardial salvage. Therefore, in patients with first-time AMI successfully treated with percutaneous coronary intervention (PCI), we aimed to investigate whether circulating levels of high-sensitivity C-reactive protein (hs-CRP) and PIIINP are elevated in patients with SDB compared to patients without SDB. Methods and Results: This cross-sectional analysis included a total of 88 eligible patients with first AMI and PCI pooled from two prospective studies and stratified according to the apnea–hypopnea index (AHI, with SDB: AHI ≥ 15 h^−1^). We analyzed circulating levels of hs-CRP and PIIINP 3–5 days after PCI. Patients with SDB had significantly higher levels of hs-CRP (18.3 mg/L [95% CI, 8.0–42.6] vs. 5.8 mg/L [95% CI, 4.2–19.8], *p* = 0.002) and PIIINP (0.49 U/mL [95% CI, 0.40–0.60] vs. 0.33 U/mL [95% CI, 0.28–0.43], *p* < 0.001). In a multivariable linear regression model accounting for important clinical confounders, SDB significantly predicted circulating levels of hs-CRP (*p* = 0.028). Similarly, only SDB was independently associated with PIIINP (*p* < 0.001). Only obstructive but not central AHI correlated with circulating levels of hs-CRP (*p* = 0.012) and PIIINP (*p* = 0.006) levels. Conclusions: The presence of obstructive SDB after AMI was independently associated with increased circulating levels of hs-CRP and PIIINP. Our results emphasize the important role of SDB as a common comorbidity and indicate increased inflammation and myocardial fibrosis in these patients.

## 1. Introduction

Following acute myocardial infarction (AMI), myocardial stretch due to increased mural stress at the infarct site activates a cascade of processes in myocytes and the extracellular matrix. The necrotic tissue activates an inflammatory response and attracts neutrophils and macrophages that eliminate cell debris and produce more pro-inflammatory cytokines [1]. Subsequently, resident cardiac fibroblasts transform into myofibroblasts producing collagen and α-smooth muscle actin [2]. The invasion of various immune cells, the changes in cell metabolism due to impaired oxygen diffusion, the activation of the sympathetic nervous system and the renin–angiotensin–aldosterone axis lead to an orchestrated interplay that ultimately results in myocardial fibrosis. Interestingly, early collagen deposition is thought to be reversible and may even prevent myocardial rupture [1,2]. Definite irreversible replacement fibrosis appears after 3 months and is associated with adverse left ventricular remodeling, arrhythmias, and the risk of developing heart failure [1].

There are two main types of collagens in the heart, collagen I and collagen III, both responsible together with elastin for the mechanical properties of the interstitial tissue. Collagen III fibrils provide, as opposed to collagen I, more elastic characteristics and are more specific to the heart [3,4]. Procollagen III type aminoterminal propeptide is an extension peptide of procollagen type III, originating during the final stages of collagen synthesis, and is detectable in the human serum [5]. Therefore, the serum levels of PIIINP can be used as a measure of collagen type III synthesis. Following acute myocardial infarction, collagen turnover and collagenase activity are enhanced [6,7]. Exaggerated collagenolysis at the infarct size measured with circulating PIIINP has been shown to be linked to unfavorable cardiac remodeling and worse clinical outcomes [7,8]. Moreover, in large-scale studies, PIIINP has been already shown to be associated with cardiovascular disease, to predict the incidence of heart failure, and was proposed to provide a non-invasive assessment of fibrosis and its severity [9,10,11].

Sleep-disordered breathing (SDB) is highly prevalent in patients with first-time AMI, reaching up to two thirds [12], and is associated with increased risk of coronary heart disease, cardiac death, stroke, and all-cause mortality [13,14]. It is characterized by intermittent hypoxia, arousals from sleep, increased cardiac workload, and left ventricular mural pressure resulting in less myocardial salvage and greater infarct size [12]. Patients with SDB after AMI are at risk of developing left ventricular dysfunction and left ventricular remodeling [13,15]. The underlying biological pathways are not fully understood. The emerging body of evidence suggests that increased inflammation, dysregulation of sympathetic activation, vascular endothelial dysfunction, and oxidative stress are major contributors to the disease progression [13,15].

Inflammatory processes affect the tissue re-arrangement at the infarct site after AMI and, when excessive, lead to adverse cardiac remodeling. We hypothesize that SDB favors inflammation and myocardial fibrosis resulting in less myocardial salvage. Therefore, we sought to investigate in patients with first-time AMI successfully treated with percutaneous coronary intervention whether levels of circulating high-sensitivity C-reactive protein (hs-CRP) and PIIINP are elevated if SDB is present.

## 2. Materials and Methods

### 2.1. Study Design

For this cross-sectional analysis, we included patients from two prospective clinical studies. The first study was a prospective observational study conducted at University Hospital Regensburg (Regensburg, Germany) between March 2009 and March 2012 [12]. The second, TEAM-ASV I (Treatment of Sleep Apnea Early After Myocardial Infarction With Adaptive Servo-Ventilation Trial I, NCT02093377), was a multicentric, randomized, open-label, parallel study with a blinded assessment of outcomes with the recruitment of participants between February 2014 and August 2020 [16]. The primary aim of TEAM-ASV I was to compare treatment with PCI and optimal medical therapy versus PCI, optimal medical therapy, and adaptive serve ventilation (ASV) in patients with AMI and SDB. In contrast to the prospective observational study, TEAM-ASV I investigated only patients with SDB, leading to an oversampling of SDB patients [12,16]. Both studies had identical inclusion criteria: age 18–80 years, first AMI, ST elevation in ECG or acute occlusion of the coronary artery, and primarily successful PCI achieved <24 h after the onset of symptoms. The study protocols were reviewed and approved by the local institutional ethics committee and are in accordance with the Declaration of Helsinki. Individual written informed consent was obtained before the start of the studies.

### 2.2. Blood Work

Venous blood samples were collected during the first 2 h after waking up in the morning 3 to 5 days after PCI and before treatment with ASV. The measurement of hs-CRP and PIIINP was performed directly after blood sampling in an assigned central laboratory [16]. Levels of hs-CRP were quantified with a nephelometric analyzer (Atellica Neph630, Siemens Healthineers AG, Forchheim, Germany).

Radioimmunoassay (RIA-gnost^®^ PIIIP, Cisbio Bioassays, Codolet, France) was used to measure PIIINP. Reference values according to Cisbio Bioassays ranged from 0.3 to 0.8 U/mL. In addition, 7 patients were analyzed in an external laboratory using an ELISA kit (Cisbio Bioassays), due to the discontinuation of the radioimmunoassay method in one center. To obtain the values in the unit of the enzyme’s catalytic activity (U/mL), the values measured with the ELISA kit (μg/L) were divided by factor 8 according to the manufacturer’s recommendation.

### 2.3. Assessment of Sleep-Disordered Breathing

The assessment of SDB was performed via polysomnography in the prospective observational study (Alice System, Respironics, Pittsburgh, PA, USA) or polygraphy in the TEAM-ASV I trial (SOMNOscreenTM plus RC, SOMNOmedics, Randersacker, Germany) within 3 days after PCI, respectively. Details of the sleep recordings have been described previously [12,16]. The evaluation of the patient’s time in bed was analyzed based on body position, light detection, and a patient marker. Sleep stages, arousals, and apneas and hypopneas were determined according to the American Academy of Sleep Medicine 2012 criteria by one experienced sleep technician blinded to the clinical data [17]. SDB was defined arbitrarily as an apnea–hypopnea index (AHI) ≥ 15 events per hour (total recording time). CSA was defined as cAHI/ AHI > 50% and OSA as cAHI/AHI ≤ 50%.

### 2.4. Statistical Analysis

Baseline characteristics of the pooled population were summarized according to SDB. Continuous variables were compared using Student’s *t*-test, or the Mann–Whitney U test conforming to a normal distribution and expressed as means ± standard deviation (SD) or median with interquartile range (IQR). Nominal variables were assessed via the chi-squared test or Fisher’s exact test based on the number of observations and reported as frequencies and percentages of each category. Univariable linear regression models were applied to determine the relationship between hs-CRP, or PIIINP, and other important clinical variables. Only variables with *p* values less than 0.1 were included in the multivariable linear regression model [18]. Spearman’s correlation was applied when the data were non-normally distributed. The Spearman correlation coefficient (r_s_) indicates an association of ranks: 1 = perfect positive association; 0 = no association; and −1 = perfect negative association [19]. We interpreted the effect size according to Cohen’s convention: 0.1 = small effect; 0.3 = moderate effect; and 0.5 = large effect [20]. All reported *p* values were two-sided and the threshold for significance was set at *p* < 0.05. Statistical analysis was performed in SPSS (SPSS Statistics for Mac OS, Version 29.0, IBM Corp.: Armonk, NY, USA) and GraphPad Prism (Version 9.51 for Windows, GraphPad Software, La Jolla, CA, USA).

## 3. Results

### 3.1. Patient Population

**Figure 1:**
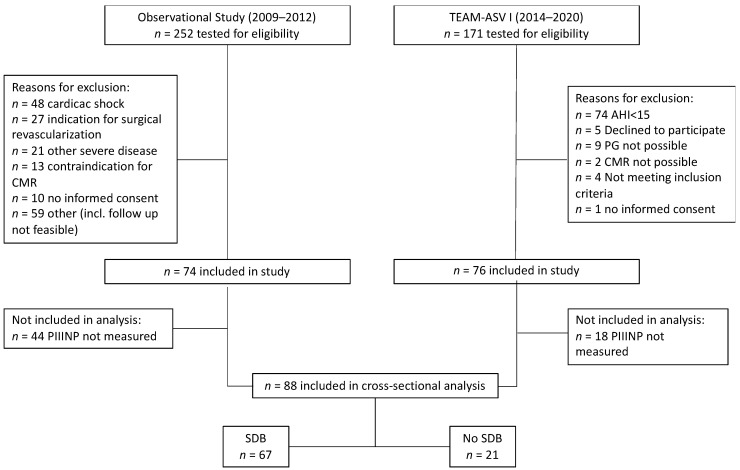
Flow diagram of patients included in the study. AHI: apnea–hypopnea index; CMR: cardiac magnetic resonance imaging; PG: polygraphy; PIIINP: procollagen III type aminoterminal propeptide; SDB: sleep-disordered breathing; TEAM-ASV I: Treatment of Sleep Apnea Early After Myocardial Infarction With Adaptive Servo-Ventilation Trial I.

**Figure 2:**
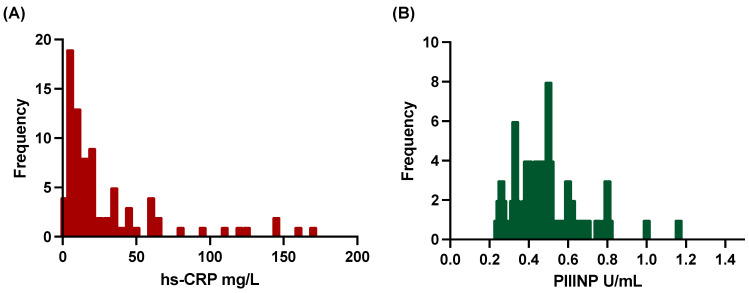
Histogram of (**A**) high-sensitivity C-reactive protein (hs-CRP) and (**B**) procollagen III type aminoterminal propeptide (PIIINP).

The data set covers two patient populations with a total number of 150 patients. Levels of circulating PIIINP obtained after study enrollment were available from 88 of these patients (mean age 57 ± 10 years; 16% female) (Figure 1 and Figure 2). The patients were stratified into two groups according to AHI: AHI ≥ 15 h^−1^ with SDB (*n* = 67) and AHI < 15 h^−1^ without SDB (*n* = 21). The median AHI in the SDB group was 25.8 h^−1^. Patients with SDB were older, had higher BMI and worse renal function (Table 1). Of note, creatine kinase, the pain-to-balloon time, and LVEF were similar in both groups. 

### 3.2. hs-CRP and PIIINP Correlate with SDB

**Figure 3:**
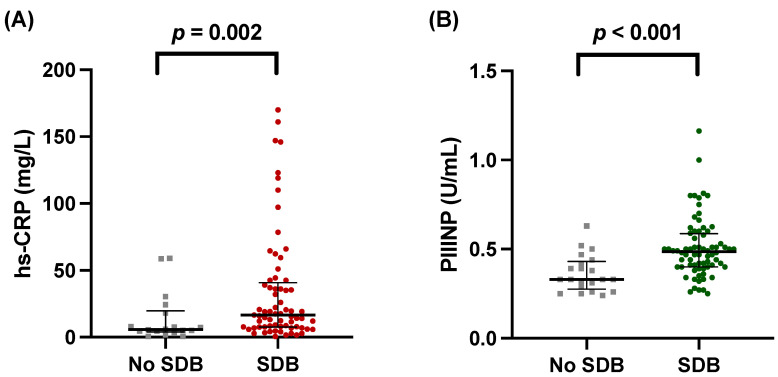
Circulating levels of high-sensitivity C-reactive protein (hs-CRP) and procollagen III type aminoterminal propeptide (PIIINP) according to the presence of sleep disorder breathing (SDB). (**A**) hs-CRP (18.3 mg/L [95% CI, 8.0–42.6] vs. 5.8 mg/L [95% CI, 4.2–19.8], *p* = 0.002) and (**B**) PIIINP (0.49 U/mL [95% CI, 0.40–0.60] vs. 0.33 U/mL [95% CI, 0.28–0.43], *p* < 0.001).

**Figure 4:**
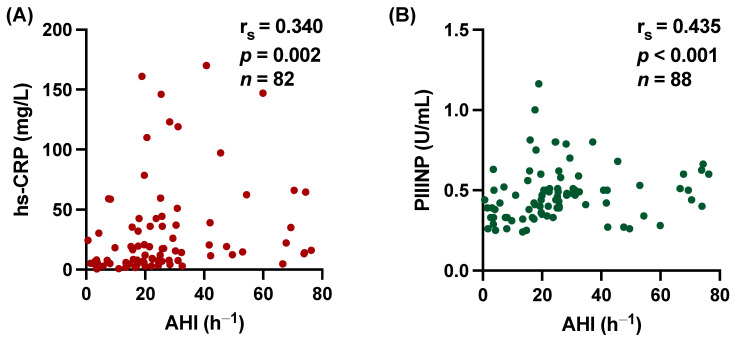
Scatter plot demonstrating the significant relations between high-sensitivity C-reactive protein (hs-CRP) and procollagen III type aminoterminal propeptide (PIIINP) with apnea–hypopnea index (AHI). (**A**) hs-CRP (r_s_ = 0.340, 95% CI 0.145 to 0.538, *p* = 0.002) and (**B**) PIIINP (r_s_ = 0.435, 95% CI 0.105 to 0.494, *p* < 0.001). r_s_ = Spearman correlation coefficient; CI: confidence interval.

We found that both levels of hs-CRP and PIIINP were significantly higher in patients with SDB. For hs-CRP 18.3 [95% CI, 8.0–42.6] vs. 5.8 mg/L [95% CI, 4.2–19.8], *p* = 0.002 (Figure 3A). For PIIINP 0.49 U/mL [95% CI, 0.40–0.60] vs. 0.33 U/mL [95% CI, 0.28–0.43], *p* < 0.001 (Figure 3B). Scatter plots also demonstrate the significant correlation between hs-CRP and AHI (r_s_ = 0.340, *p* = 0.002, Figure 4A) and PIIINP and AHI (r_s_ = 0.435, *p* < 0.001, Figure 4B) with moderate effect size. In accordance, the time with oxygen saturation <90% (T90) showed significant correlation with hs-CRP levels (r_s_ = 0.245, *p* = 0.028), and the oxygen desaturation index (ODI) showed a strong but not quite significant correlation with hs-CRP (r_s_ = 0.198, *p* = 0.079). No significant correlation was found between PIIINP and T90 (r_s_ = 0.169, *p* = 0.117) or ODI (r_s_ = 0.157, *p* = 0.153).

### 3.3. Central vs. Obstructive Sleep Apnea

**Figure 5:**
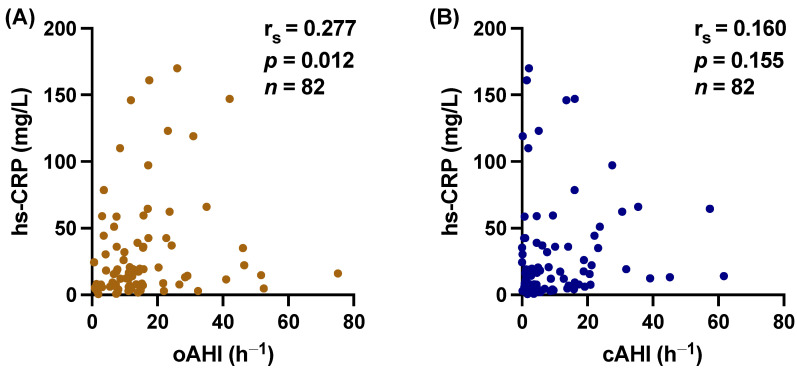
Scatter plot demonstrating the significant relations between high-sensitivity C-reactive protein (hs-CRP) and (**A**) obstructive apnea–hypopnea index (oAHI) (r_s_ = 0.277, 95% CI 0.058 to 0.470, *p* = 0.012), but not (**B**) central apnea–hypopnea index (cAHI) (r_s_ = 0.160, 95% CI −0.062 to 0.366, *p* = 0.155). r_s_ = Spearman correlation coefficient; CI: confidence interval.

**Figure 6:**
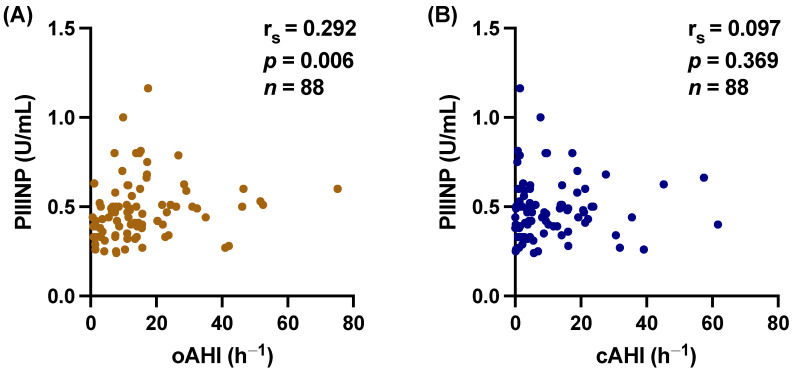
Scatter plot demonstrating the significant relations between procollagen III type aminoterminal propeptide (PIIINP) and (**A**) obstructive apnea–hypopnea index (oAHI) (r_s_ = 0.292, 95% CI 0.082 to 0.477, *p* = 0.006), but not (**B**) central apnea–hypopnea index (cAHI) (r_s_ = 0.097, 95% CI −0.116 to 0.302, *p* = 0.369). r_s_ = Spearman correlation coefficient; CI: confidence interval.

Additionally, we tested separately for the obstructive apnea–hypopnea index (oAHI) and the central apnea–hypopnea index (cAHI), which showed a significant correlation of hs-CRP with oAHI (r_s_ = 0.277, *p* = 0.012) but not with cAHI (r_s_ = 0.160, *p* = 0.155) (Figure 5). Similarly, PIIINP correlated with oAHI (r_s_ = 0.292, *p* = 0.006), whereas cAHI did not (r_s_ = 0.097, *p* = 0.369) (Figure 6).

### 3.4. Regression Analysis

To test for clinical comorbidities, univariable linear regressions for circulating levels of hs-CRP and PIIINP were performed. Table 2 and Table 3 show the relationship of hs-CRP and PIIINP with important clinical confounders such as age, gender, body mass index, current smoking, arterial hypertension, diabetes mellitus, peripheral artery disease, LVEF, or eGFR. For hs-CRP, only SDB and LVEF met the α-level of <0.1 and were further calculated in a multivariable linear regression model. Importantly, in multivariable linear regression, only SDB and LVEF remained significant predictors for circulating levels of hs-CRP (*p* = 0.028 and *p* = 0.005, respectively, Table 2). For circulating levels of PIIINP, only the presence of SDB and age met the significance criterion for the multivariable linear analysis (*p* < 0.1), from which only SDB was an independent modulator of circulating levels of PIIINP (*p* < 0.001, Table 3).

## 4. Discussion

The main and novel finding of this study is that circulating levels of hs-CRP and PIIINP were significantly higher in patients with first-time AMI when SDB was present. This association remained significant after adjusting for clinically important co-variables. Our data suggest that the presence of SDB may have a detrimental effect on myocardial fibrosis associated with exaggerated inflammation. Furthermore, obstructive but not central AHI were accompanied by higher levels of PIIINP.

Our data showed a significant association of higher hs-CRP levels with SDB, indicating a stronger inflammatory response. Not only higher hs-CRP levels but also the presence of other cytokines (e.g., interleukin 6, tumor necrosis factor α) and adhesion molecules (e.g., intercellular adhesion molecule-1, vascular cell adhesion molecule-1, selectins) have been detected in patients with SDB [21]. Ultimately, inflammatory cytokines activate pro-fibrotic signaling cascades exaggerated in SDB [21]. Importantly, SDB has been shown to be associated with greater infarct expansion and impaired healing depicted with less myocardial salvage [12]. One possible explanation may be that enhanced collagen synthesis results in extended myocardial fibrosis denoted with more collagen deposition. Our results support this hypothesis, since hs-CRP and PIIINP levels were independently associated with SDB. This finding strengthens further the role of inflammation featured in SDB.

A previous study of Fisser et al. investigated the association of SDB and left ventricular remodeling after AMI and found that spheric cardiac remodeling was associated with obstructive but not central sleep apnea [22]. In our study, obstructive AHI was significantly correlated with circulating levels of PIIINP. Conversely, central AHI did not show any association. Our data suggest that increased left ventricular transmural pressure occurring in patients with OSA may play an important role in myocardial fibrosis and cardiac remodeling.

The current literature has failed to show any relevant benefit of continuous positive airway pressure (CPAP) therapy on inflammatory markers or any increase after CPAP withdrawal [23,24]. For example, the RICCADSA trial could not determine any significant effect of CPAP on long-term adverse cardiovascular outcomes or inflammatory markers in patients with coronary artery disease [24,25]. This may be due to the vicious circle of inflammation that occurs in irreversibly damaged tissue after years of exposure to reactive oxygen species caused by intermittent hypoxia and sympathetic activation. However, both intermittent hypoxia and sympathetic activation were shown to be influenced by CPAP [23]. Therefore, one can speculate that perhaps an early treatment could break the vicious inflammatory circle. Interestingly, the TEAM-ASV I trial investigated the impact of adaptive servo-ventilation (ASV) on myocardial salvage in patients with SDB and first-time AMI. Arzt et al. has shown that the percentage reduction in infarct size at 12 weeks in the ASV group was more than double then in the control group [26]. From this perspective, further studies such as TEAM-ASV I are warranted to elucidate the effect of positive airway pressure after the acute onset of inflammation such as AMI.

Furthermore, novel pharmacotherapeutic approaches to improve adverse remodeling are currently being investigated. Intravenous administration of hyaluronan oligosaccharide and the use of human recombinant collagen III hydrogel both offer promising potential as therapeutics [27,28,29]. Also, a variety of other medications such as pirfenidone, angiotensin receptor blockers, or NLRP3 inflammasome inhibitors are currently evaluated in clinical trials [30]. Additional strategies for anti-fibrotic therapies comprise miRNAs, epigenetic regulation, CRISPR technology, or lipid nanoparticle-based transfer of ribonucleic acid [1,30]. Greater insight into important clinical comorbidities such as SDB could help to select suitable patients who might better benefit from the anti-fibrotic treatment.

Several limitations need to be considered when interpreting the results of this study. Firstly, the retrospective manner of the present analysis caused a noticeable loss of patients where no data on PIIINP were available. Additionally, it was not possible to evaluate the circulating levels of blood markers at follow-up due to the randomization of patients for ASV treatment in the TEAM-ASV I trial. Also, the interaction between SDB and AMI could not be clearly determined because we did not include a control group without AMI. Secondly, the circulating levels of PIIINP in seven patients were quantified using a different method. Thirdly, the sleep characteristics in the prospective observational study were assessed using polysomnography and in the TEAM-ASV I with polygraphy. Therefore, the sleep characteristics by polysomnography were calculated based on the total recording time and not the total sleep time. Lastly, despite the multicentric properties of this pooled dataset, the total number of patients in the group without SDB is quite small, which limits the statistical power.

## 5. Conclusions

Our main novel finding is that circulating levels of hs-CRP and PIIINP were independently associated with SDB in patients with AMI. Since it has been shown that patients with SDB show impaired myocardial healing after AMI [12], SDB-related inflammation and fibrosis may complicate the course of ACS in these patients. Moreover, we show here that obstructive but not central apneas were associated with abnormal hs-CRP and PIIINP levels, suggesting that airway obstruction may be involved in the pathophysiology. Further studies are warranted to confirm the role of SDB on cardiac remodeling.

## Figures and Tables

**Table 1:** Baseline characteristics.

	All Patients *n* = 88	No SDB *n* = 21	SDB *n* = 67	*p* Value
Age, years	57 (±10)	52 (±9)	58 (±9)	0.011
Female gender, *n* (%)	16 (18)	4 (19)	12 (18)	0.100
BMI, kg/m^2^	30 (±5)	27 (±2)	30 (±5)	0.011
Arterial hypertension, *n* (%)	48 (55)	10 (48)	38 (57)	0.456
Diabetes mellitus, *n* (%)	15 (17)	2 (10)	13 (19)	0.506
Peripheral artery disease, *n* (%)	4 (5)	1 (5)	3 (5)	1.000
Current smoker, *n* (%)	43 (49)	12 (60)	31 (46)	0.281
AHI, h^−1^	23.2 (15.3–32.0)	4.3 (3.3–9.0)	25.8 (20.0–41.7)	<0.001
oAHI, h^−1^	11.9 (6.8–17.5)	2.8 (1.4–6.6)	14.6 (10.5–23.2)	<0.001
cAHI, h^−1^	5.1 (1.5–16.1)	2.0 (0.7–4.4)	9.3 (2.6–18.9)	<0.001
ODI, h^−1^	15.5 (6.7–24.7)	3.7 (2.3–6.7)	18.9 (12.4–28.9)	<0.001
T90%, % TRT	2.4 (0.43–15.5)	0.5 (0.2–15.8)	4.7 (0.6–15.5)	0.139
LVEF, %	52 (±9)	50 (±8)	52 (±9)	0.492
LVEDV index, mL/m^2^	76 (67–87)	74 (64–84)	76 (65–83)	0.192
LVESV index, mL/m^2^	37 (28–50)	37 (28–50)	37 (28–50)	0.903
STEMI, *n* (%)	74 (86)	21 (100)	53 (82)	0.034
Multivessel disease, *n* (%)	44 (50)	10 (48)	34 (51)	0.803
Pain-to-balloon time, h	4.2 (2.8–10.1)	6.8 (2.8–17.8)	4.0 (2.8–8.5)	0.132
NT-proBNP max, pg/mL	926 (314–1598)	608 (291–1766)	936 (337–1536)	0.841
CK, U/L	218 (130–358)	240 (186–396)	200 (118–358)	0.129
Creatinine, mg/dL	0.91 (0.80–1.04)	0.92 (0.74–1.02)	0.91 (0.80–1.04)	0.038
eGFR, mL/min/1.73 m^2^	79 (±23)	96 (±15)	74 (±23)	<0.001
LDL, mg/dL	127 (±31)	131 (±26)	126 (±33)	0.511
Leukozyten, 10^9^/L	10.1 (±3.2)	12 (±3.0)	9.6 (±3.0)	0.004
Hemoglobin, g/dL	14.4 (±1.4)	15.0 (±1.1)	14.2 (±1.5)	0.009
Thrombozyten, 10^9^/L	223 (203–284)	236 (199–291)	236 (204–276)	0.858

AHI: apnea–hypopnea-index; BMI: body mass index; cAHI: central apnea–hypopnea-index; CK: creatine kinase; eGFR: estimated glomerular filtration rate; LDL: low density lipoprotein; LVEDV: left ventricular end-diastolic volume: LVEF: left ventricular ejection fraction; LVESV: left ventricular end-systolic volume; NT-proBNP: N-terminal pro-B-type natriuretic peptide; oAHI: obstructive apnea–hypopnea-index; ODI: oxygen desaturation index; SDB: sleep-disordered breathing; STEMI: ST-elevation myocardial infarction; T90: time with oxygen saturation <90%; TRT: total recording time. Values are expressed as means ± 95 standard deviation (SD), median with interquartile range (IQR), or frequencies and percentages of each category.

**Table 2:** Univariable and multivariable linear regression of high-sensitivity C-reactive protein (hs-CRP).

	Univariable Linear Regression Analysis			Multivariable Linear Regression Analysis		
hs-CRP [mg/L]	B	95% CI	*p* Value	B	95% CI	*p* Value
SDB	21.961	1.574 to 42.349	0.035	21.510	2.397 to 40.623	0.028
Age, years	0.318	−0.618 to 1.255	0.501			
Female sex	−2.125	−24.017 to 19.767	0.847			
BMI	−10.697	−33.637 to 12.242	0.356			
Current smoker	5.674	−11.807 to 23.156	0.520			
Arterial hypertension	10.350	−6.984 to 27.684	0.238			
Diabetes mellitus	−0.052	−0.147 to 0.043	0.280			
PAD	−26.425	−66.279 to 13.428	0.191			
LVEF, %	−1.151	−2.046 to −0.255	0.013	−1.262	−2.138 to −0.386	0.005
eGFR, mL/min/1.73 m^2^	0.021	−0.357 to 0.400	0.911			

B: unstandardized linear regression coefficient; BMI: body mass index; CI: confidence interval; LVEF: left ventricle ejection fraction; eGFR: estimated glomerular filtration rate; hs-CRP: high-sensitivity C-reactive protein; PAD: peripheral artery disease.

**Table 3:** Univariable and multivariable linear regression of procollagen type III aminoterminal peptide (PIIINP).

	Univariable Linear Regression Analysis			Multivariable Linear Regression Analysis		
PIIINP [U/mL]	B	95% CI	*p* Value	B	95% CI	*p* Value
SDB	0.149	0.071 to 0.227	<0.001	0.146	0.065 to 0.227	<0.001
Age, years	0.003	−0.001 to 0.007	0.089	0.001	−0.002 to 0.005	0.437
Female sex	−0.003	−0.096 to 0.091	0.954			
BMI	0.003	−0.005 to −0.010	0.484			
Current smoker	−0.047	−0.119 to 0.025	0.195			
Arterial hypertension	−0.026	−0.098 to 0.046	0.472			
Diabetes mellitus	−0.052	−0.147 to 0.043	0.280			
PAD	−0.055	−0.227 to 0.118	0.531			
LVEF, %	0.001	−0.003 to 0.005	0.512			
eGFR, mL/min/1.73 m^2^	0.001	−0.001 to 0.002	0.394			

B: unstandardized linear regression coefficient; BMI: body mass index; CI: confidence interval; LVEF: left ventricle ejection fraction; eGFR: estimated glomerular filtration rate; PIIINP: procollagen type III aminoterminal peptide; PAD: peripheral artery disease.

## Data Availability

The data underlying this article will be shared upon reasonable request to M.A. The data are not publicly available due to privacy restrictions.
